# Early Respiratory Mechanics Alterations in Smokers: Correlative Analysis of the Forced Oscillation Technique and Computerized Spirometry

**DOI:** 10.7759/cureus.76677

**Published:** 2024-12-31

**Authors:** Atul K Sharma, Smita R Sorte, Sachin B Rathod, Mrunal Phatak

**Affiliations:** 1 Physiology, All India Institute of Medical Sciences, Nagpur, Nagpur, IND

**Keywords:** copd: chronic obstructive pulmonary disease, forced oscillation technique, impulse oscillometry, reactance, resistance, smoker, spirometry

## Abstract

Introduction: Smoking remains a significant public health concern, associated with a myriad of diseases, including chronic obstructive pulmonary disease, cardiovascular diseases, and various cancers. While spirometry is the gold standard for assessing lung function, it may lack sensitivity in detecting early changes in respiratory mechanics induced by smoking. This study aims to evaluate the clinical potential of the forced oscillation technique (FOT) in detecting early changes in the respiratory mechanics of smokers and its correlation with computerized spirometry.

This study aims to bridge this gap by investigating the utility of FOT, a noninvasive method requiring less patient cooperation, in detecting early changes in respiratory mechanics among smokers.

Method: The study was conducted on 36 healthy nonsmokers and 36 healthy smokers. We conducted FOT and computerized spirometry, comparing these parameters between the two groups and finding correlations between FOT and spirometry parameters.

Result: A statistically significant difference was found across different smoking indices for computerized spirometer parameters forced vital capacity (FVC) and forced expiratory volume in one second (FEV1), marginal significance for peak expiratory flow, and no statistically significant for FEV1/FVC ratio and forced expiratory flow between 25 and 75% of vital capacity. We did not find statistically significant differences across different smoking indexes for FOT parameters, resistance at 5 Hz (R5), resistance at 20 Hz (R20), resistance difference between 5 and 20 Hz (R5-20), reactance at 5 Hz, reactance at 20 Hz (X20), area under the reactance curve (AX), resonance frequency (Fres), and resistance at resonance. However, Fres had shown marginal significance. The study revealed notable negative correlations between airway resistance parameters (such as R5 and R5-20) and the FEV1/FVC ratio, and significant negative correlations were also identified between airway reactance parameters (AX and Fres) and FEV1.

Conclusion: The results emphasize that FVC and FEV1 appear to be early markers of damage. The smoking index (1-70) did not significantly influence the FOT parameters in the examined population with the Quark i2m FOT machine (COSMED, Italy). Nevertheless, marginal significance in FOT Fres parameters suggests potential alterations with longer smoking durations, warranting further investigation. Also, significant negative correlations were found between the FOT and pulmonary function test parameters.

## Introduction

Smoking is associated with an increased risk of about more than 60 disease diagnoses and is a predominant cause of many common diseases, including cancer, cardiovascular disease, chronic bronchitis, and chronic obstructive pulmonary disease (COPD) [1]. Many studies have shown the negative effect of cigarette smoking on pulmonary function tests (PFTs) on spirometry [2-7]. Smoking is a leading cause of COPD and is associated with changes in respiratory mechanics [8]. Early detection of these changes is important to prevent the development of COPD. Spirometry is not sensitive enough to detect early lung function abnormalities [9]. It requires good subject cooperation and maximal effort and may be unreliable and variable if suboptimal maneuvers are performed [10].

The forced oscillation technique (FOT) is a method for noninvasively assessing respiratory mechanics: resistance and reactance during spontaneous breathing, which demands little patient cooperation [11]. One of the most remarkable features of FOT to spirometry is that it has much greater sensitivity to detect peripheral airway obstruction. Few studies have evaluated the usefulness of FOT in detecting early smoking-related changes in the lungs, but there is no agreement on the findings of these studies regarding the sensitivity and reliability of different parameters to detect small airway abnormalities in smokers, and the studies did not use comparable oscillation techniques [8]. However, more research is needed to fully evaluate the clinical potential of FOT in smokers and its correlation with computerized spirometry.

The study can bridge the gap in the early detection of respiratory abnormalities in smokers. Currently, spirometry is the standard diagnostic tool for respiratory diseases, but it may not be sensitive enough to detect early changes in respiratory mechanics. By evaluating the clinical potential of FOT in detecting early changes in the respiratory mechanics of smokers, the study can provide a more comprehensive assessment of respiratory function and enable early detection of respiratory abnormalities in smokers from the Indian population. Additionally, the correlation between FOT and spirometry parameters suggests that these two methods may be used together to provide a more comprehensive assessment of lung function in smokers.

## Materials and methods

The study was designed as an observational, cross-sectional study and conducted in the Pulmonary Function Test Lab, Department of Physiology, All India Institute of Medical Sciences, Nagpur. The study participants were patients or their relatives visiting the screening area and Pulmonary Outpatient Department, aged between 18 and 60 years. Participant selection employed a simple randomized, convenience sampling method due to the limited time available for the study, as stipulated by the Indian Council of Medical Research guidelines, which set two months for the study.

Inclusion criteria for the study required participants to be willing to participate and comprised healthy subjects aged 18-60 years who were nonsmokers (never smoked) and had no history of tobacco use, active tuberculosis (TB), or respiratory infection. Participants were excluded if they were on bronchodilators, had recent eye surgery, experienced chest pain, had a recent heart attack or unstable heart condition, were pregnant, had stomach bloating or diarrhea, suffered from thoracic, abdominal, or cerebral aneurysm, experienced dizziness, had a history of neuromuscular disorders affecting respiratory muscles, had hypertension, or had a history of occupational exposure.

Similarly, smokers aged 18-60 years with a history of smoking for a minimum of one year were included, provided they had no history of active TB or respiratory infection, were not on bronchodilators, had no recent eye surgery, chest pain, recent heart attack, or unstable heart condition, were not pregnant, and had no stomach bloating or diarrhea, thoracic, abdominal, or cerebral aneurysm, dizziness, history of neuromuscular disorders affecting respiratory muscles, hypertension, or occupational exposure history. The smoking index, calculated as the number of cigarettes smoked per day multiplied by the duration of years of tobacco use, was used to select smokers with a smoking index between 1 and 70 for inclusion in the study.

Participants were excluded if they were unwilling to participate, were former smokers who had smoked fewer than 100 cigarettes in their lifetime but had quit smoking during the past year [12], or had any history of tobacco use, active TB, or respiratory infection, were on bronchodilators, had recent eye surgery, experienced chest pain, had a recent heart attack or unstable heart condition, were pregnant, had stomach bloating or diarrhea, suffered from thoracic, abdominal, or cerebral aneurysm, experienced dizziness, had a history of neuromuscular disorders affecting respiratory muscles, had hypertension, or had a history of occupational exposure.

The study was conducted for two months, with a sample size comprising 36 smokers and 36 nonsmokers, totaling 72 participants. The sample size calculation was based on the variable resistance at 5 Hz, R5 (cm H_2_O/L/s), representing the total resistance of the respiratory system. Data from a study on smokers showed a standard deviation of 0.75 cm H_2_O/L/s. The calculation aimed to detect a minimum difference of 0.5 cm H_2_O/L/s with 80% power and a 5% two-tailed significance level, resulting in 36 individuals per group as a sufficient sample size [13].

The study was approved by the Institutional Research Committee and Ethics Committee, letter no. IEC/Pharmac/2023/664 dated October 31, 2023. Participants were recruited from hospitals and college campuses. The participants willing to participate and fulfill the inclusion criteria were selected for the study. They were informed about the aims, objectives, procedures, risks, and benefits of the study. Participants were asked to sign the informed consent form and fill out the participant information sheet.

The appointment was given, and subjects were requested to visit the PFT laboratory in the Department of Physiology to perform FOT and computerized spirometry in a single visit. According to American Thoracic Society guidelines, they were informed to avoid intake of caffeine-containing products (tea, coffee, and cola) for at least six hours, alcohol for at least four hours, large meals for at least two hours, avoid smoking for at least one hour, and vigorous exercise for at least 30 minutes before the procedure. Subjects were informed to wear comfortable clothes that allow full expansion of the chest and abdomen [9].

FOT was performed before computerized spirometry, as the forced maneuvers of spirometry impact the resistance and reactance values of FOT [14]. The FOT was conducted using the Quark i2m FOT machine (COSMED, Italy). Before the test, the FOT procedure was explained thoroughly to the patient. The test was administered with the patient seated comfortably, ensuring their legs were uncrossed to minimize extrathoracic pressure [14].

A nose clip was applied to prepare for the test, and the patient's mouthpiece was adjusted to a comfortable height, allowing the neck to be slightly extended. A tight seal between the mouthpiece and the patient's lips was ensured to prevent any air leakage. Additionally, the cheeks were held firmly either by the patient with their hands or by an assistant pressing firmly from behind [14].

During the FOT procedure, the patient was instructed to breathe normally at functional residual capacity in a relaxed state for 10 seconds. Throughout this period, sound impulses were delivered into the lungs, from which mean reactance and resistance values were derived at frequencies ranging from 5 to 20 Hz.

A minimum of three such tests were conducted to ensure consistency and reliability of the results. The respiratory parameters obtained from the FOT included R5, resistance at 20 Hz (R20), the resistance difference between 5 and 20 Hz (R5-20), reactance at 5 Hz (X5), reactance at 20 Hz (X20), reactance area, resonant frequency (Fres), and resistance at resonance R(Fres).

The spirometry procedure was conducted using the NDD spirometry "Easy in PC" model version 3.9.2.0. Before the test, the patient was briefed about the forced vital capacity (FVC) procedure. The test was administered with the patient seated comfortably. To ensure accuracy, a nose clip was applied, and the connection between the mouthpiece and the patient's lips was tightly sealed to prevent any air leakage.

The patient was instructed to breathe exclusively through the mouthpiece for six normal breaths, with no interaction with ambient air. Following this, they were directed to perform a rapid, maximal inhalation without pausing at the end of inspiration, followed by a forceful exhalation lasting at least six seconds.

Throughout the procedure, the patient was encouraged to exert their maximum effort to obtain the most accurate results. Three repeatable maneuvers were performed, and the best result from these attempts was recorded for analysis.

The respiratory parameters obtained included forced expiratory volume in the first second (FEV1), FVC, FEV1/FVC ratio, peak expiratory flow (PEF), and forced expiratory flow between 25% and 75% of FVF (FEF25-75).

Statistical analysis

The data were analyzed using the Statistical Software Jamovi 2.8.28, jamovi (Version 2.3) (Computer Software). Retrieved from https://www.jamovi.org, solid version [15], and appropriate descriptive and inferential statistical techniques were used. The descriptive analytical techniques consist of tables and numerical summary measures such as central tendency and dispersion.

Normality for computerized spirometry parameters and FOT parameters was tested using the Q-Q plot and was found to be normally distributed. A one-way analysis of variance (ANOVA) was used to compare the computerized spirometry respiratory parameter and FOT respiratory mechanics parameter with the smoking index. The significance level was set at p ≤ 0.05. A Pearson correlation matrix and heat map were generated between PFT and FOT parameters.

## Results

There were no statistically significant differences in weight, and body mass index between nonsmokers and smokers with different smoking indexes. Marginal difference was found in age and height (Table 1).

**Table 1:** Comparison of demographic parameters with smoking index using one-way ANOVA The statistical test used is one-way ANOVA (Fisher’s) p < 0.05 is considered significant SD: standard deviation; BMI: body mass index; ANOVA: analysis of variance

Variables	Smoking index	n	Mean	SD	p
Age	0	36	26.94	9.53	0.0487
	1-20	25	23.72	1.70	
	21-40	6	28.33	3.72	
	41-70	5	33.60	11.78	
Height	0	36	171.34	5.15	0.0454
	1-20	25	171.82	11.20	
	21-40	6	162.58	4.01	
	41-70	5	167.00	3.54	
Weight	0	36	68.17	11.53	0.7705
	1-20	25	69.88	11.40	
	21-40	6	64.55	11.71	
	41-70	5	67.54	11.39	
BMI	0	36	23.15	3.35	0.8524
	1-20	25	24.03	6.24	
	21-40	6	24.34	3.77	
	41-70	5	24.17	3.66	
Cigarettes smoked per day	0	36	0.00	0.00	0.001
	1-20	25	2.88	1.01	
	21-40	6	4.33	1.97	
	41-70	5	4.40	1.95	
Smoking years	0	36	0.00	0.00	0.001
	1-20	25	2.92	1.58	
	21-40	6	6.83	1.72	
	41-70	5	14.40	8.50	

The one-way ANOVA analysis was conducted on spirometry parameters, including FEV1/FVC ratio, FVC, FEV1, FEF25-75, and PEF, across different smoking indexes (0, 1-20, 21-40, and 41-70 years) and yielded mixed results. The analysis indicates that the FVC (F = 3.292, p = 0.02569^*^) and FEV1 (F = 4.142, p = 0.009^*^) are significantly affected by the smoking index, with higher indices correlating with reduced lung function as evidenced by lower FVC and FEV1 values.

The PEF values (F = 2.388, p = 0.076) did not show a statistically significant difference among the different smoking index categories; however, the marginal p values for PEF suggest a trend toward significance, indicating that higher smoking indices might be associated with a decrease in PEF values, warranting further investigation with larger sample sizes. There were no statistically significant differences observed in FEV1/FVC (F = 0.322, p = 0.809) and FEF25-75 (F = 1.475, p = 0.229), indicating that smoking history may not significantly impact these parameters within the examined groups (Table 2).

**Table 2:** Comparison of spirometry parameters with smoking index using one-way ANOVA (Fisher's) (PFT parameters) The statistical test used is one-way ANOVA ^*^p < 0.05 is considered significant FEF25-75 (L/S): forced expiratory flow between 25% and 75% of FVC (liters/second); FEV1(L): forced expiratory volume in one second (liters); FEV1/FVC: ratio of forced expiratory volume in one second to forced vital capacity; FVC (L): forced vital capacity (liters); PEF (L/S): peak expiratory flow (liters/second); PFT: pulmonary function test; ANOVA: analysis of variance

Spirometry parameters	Smoking index	n	Mean	SD	F	p
FEV1/FVC	0	36	84.57	6.857	0.322	0.80916
	1-20	25	85.52	5.139		
	21-40	6	83.56	7.854		
	41-70	5	82.92	8.464		
FVC (L)	0	36	3.98	0.584	3.292	0.02569^*^
	1-20	25	4.24	0.531		
	21-40	6	3.63	0.861		
	41-70	5	3.56	0.175		
FEV1(L)	0	36	3.36	0.564	4.142	0.00934^*^
	1-20	25	3.62	0.424		
	21-40	6	2.99	0.520		
	41-70	5	2.96	0.425		
FEF25-75 (L/S)	0	36	3.86	1.124	1.475	0.22920
	1-20	25	4.05	1.073		
	21-40	6	3.08	0.654		
	41-70	5	3.40	1.660		
PEF (L/S)	0	36	7.83	1.947	2.388	0.07650
	1-20	25	8.26	1.737		
	21-40	6	6.21	1.722		
	41-70	5	8.72	1.188		

The one-way ANOVA analysis conducted on FOT parameters across different smoking indices reveals no statistically significant differences in most parameters. Specifically, R5, R20, R5-20, X5, X20, the area under the reactance curve (AX), FRES, and R(Fres) demonstrate no significant variations across smoking duration groups, with p values above 0.05 for all comparisons. This suggests that the smoking index may not significantly influence these FOT parameters within the examined groups.

However, FRES shows a marginal p value of 0.126, indicating potential variability across the smoking index, particularly with higher mean values observed in the groups with 21-40 and 41-70 smoking Index. Further research with larger sample sizes is needed to validate any associations between smoking duration and FOT parameters (Table 3).

**Table 3:** Comparison of FOT parameters with smoking index using one-way ANOVA (Fisher's) The statistical test used is one-way ANOVA p < 0.05 is considered significant AX: area under the reactance curve; Fres: resonance frequency; R(fres): resistance at resonance; R20: resistance at 20 Hz; R5: resistance at 5 Hz; R5-20: resistance difference between 5 and 20 Hz; X20: reactance at 20 Hz; X5: reactance at 5 Hz; ANOVA: analysis of variance; FOT: forced oscillation technique

FOT parameters	Smoking index	n	Mean	SD	F	p
R5	0	36	3.662	1.385	0.950	0.421
	1-20	25	3.233	1.337		
	21-40	6	4.170	1.516		
	41-70	5	3.342	1.484		
R20	0	36	3.377	1.313	1.021	0.389
	1-20	25	2.949	0.996		
	21-40	6	3.430	0.990		
	41-70	5	2.712	1.027		
R5-20	0	36	0.204	0.754	1.064	0.370
	1-20	25	0.365	0.880		
	21-40	6	0.740	0.933		
	41-70	5	0.630	0.498		
X5	0	36	-1.501	1.568	0.484	0.695
	1-20	25	-1.373	0.832		
	21-40	6	-2.070	1.530		
	41-70	5	-1.360	0.300		
X20	0	36	0.6864	2.923	0.529	0.664
	1-20	25	4.9108	23.773		
	21-40	6	-0.2300	0.725		
	41-70	5	0.0120	0.368		
AX	0	36	8.3256	7.273	0.143	0.934
	1-20	25	8.5112	10.710		
	21-40	6	8.400	7.233		
	41-70	5	10.988	5.436		
Fres	0	36	19.0314	6.176	1.975	0.126
	1-20	25	17.7960	5.436		
	21-40	6	24.085	11.66		
	41-70	5	22.7180	7.092		
R(Fres)	0	36	3.21	1.38	0.824	0.485
	1-20	25	2.87	1.00		
	21-40	6	3.48	1.10		
	41-70	5	2.65	1.04		

The Pearson correlation matrix provides insights into the strength and direction of associations between various spirometry and FOT parameters. Each cell in the matrix represents the Pearson correlation coefficient (r), indicating the degree of the linear relationship between two variables, along with the associated p value indicating the statistical significance of the correlation. Asterisks (*) and associated p values indicate statistically significant correlations in this matrix.

The Pearson correlation matrix reveals weak-to-moderate associations between the smoking index and various spirometry and FOT parameters, as indicated by correlation coefficients (r) and associated p values. While most correlations are not statistically significant, notable relationships emerge. For instance, a significant negative correlation is observed between airway R5 and the FEV1/FVC ratio (r = -0.205, p = 0.042), suggesting that higher airway resistance is associated with lower FEV1/FVC values.

Similarly, significant negative correlations are found between the AX and FEV1 (r = -0.311, p = 0.004), as well as between Fres and FEV1 (r = -0.516, p < 0.001), indicating that increased area of reactance and resonant frequency are associated with decreased FEV1. These findings underscore potential relationships between the smoking index and respiratory function parameters, warranting further investigation into their clinical implications (Table 4).

**Table 4:** Pearson correlation matrix between spirometry and FOT parameters The statistical test used is one-way ANOVA p < 0.05 is considered significant ^*^p < 0.05, ^**^p < 0.01, ^***^p < 0.001 AX: area under the reactance curve; FEF25-75 (L/S): forced expiratory flow between 25% and 75% of FVC (liters/second); FEV1(L): forced expiratory volume in one second (liters); FEV1/FVC: ratio of forced expiratory volume in one second to forced vital capacity; Fres: resonance frequency; FVC (L): forced vital capacity (liters); PEF (L/S): peak expiratory flow (liters/second); R (fres): resistance at resonance; R20: resistance at 20 Hz; R5: resistance at 5 Hz; R5-20: resistance difference between 5 and 20 Hz; X20: reactance at 20 Hz; X5: reactance at 5 Hz; FOT: forced oscillation technique

FOT parameters	Smoking index	FEV1/FVC	FVC	FEV1	FEF25-75 (L/S)	PEF (L/S)
Smoking index	-	-0.064	-0.155	-0.177	-0.122	0.048
p value	-	0.595	0.193	0.137	0.309	0.690
R5	-0.019	-0.205^*^	-0.084	-0.183	-0.230^*^	-0.081
p value	0.875	0.042	0.484	0.062	0.026	0.500
R20	-0.143	-0.162	0.043	-0.045	-0.146	-0.139
p value	0.232	0.087	0.722	0.354	0.110	0.184
R5-20	0.200	-0.012	-0.241^*^	-0.239^*^	-0.119	0.121
p value	0.092	0.461	0.041	0.022	0.160	-0.145
X5	-0.036	0.031	-0.024	-0.006	-0.056	0.223
p value	0.765	0.602	0.844	0.482	0.319	-0.040
X20	-0.013	0.029	0.127	0.131	0.054	0.736
p value	0.915	0.596	0.288	0.864	0.675	-0.088
AX	0.099	-0.156	-0.251^*^	-0.311^**^	-0.242^*^	0.465
p value	0.408	0.095	0.033	0.004	0.020	-0.203
Fres	0.141	-0.152	-0.469^***^	-0.516^***^	-0.288^***^	0.088
p value	0.238	0.101	-	-	0.007	-0.137
R(Fres)	-0.099	-0.174	0.039	-0.052	-0.160	0.251
p value	0.408	0.072	0.743	0.331	0.090	-

## Discussion

The evaluation of early changes in respiratory mechanics induced by smoking is crucial for timely detection and intervention, given the substantial health risks associated with smoking-related respiratory diseases. Understanding these changes can facilitate the development of effective diagnostic and monitoring strategies to mitigate the impact of smoking on respiratory health. In this study, the clinical potential of the FOT in detecting early respiratory alterations in smokers is investigated, alongside its correlation with computerized spirometry parameters.

We found statistically significant differences for computerized spirometer parameters FVC and FEV1, marginal difference for PEF, no statistically significant differences for FEV1/FVC ratio, and FEF25-75 across different smoking indexes. The results emphasize that FVC and FEV1 appear to be early markers of damage.

We did not find statistically significant differences for FOT parameters R5, R20, R5-20, X5, X20, AX, Fres, and R(Fres). However, Fres showed a marginal difference. These findings suggest that the smoking index may not significantly influence the examined population with the i2m Quark COSMED FOT machine.

In contrast to our study, a study by Faria et al. conducted PFT and FOT on smokers from smoking cessation centers, including 26 COPD patients. They stratified smokers in pack-years (mean number of packs, 20 cigarettes, consumed daily by the number of years of smoking) into <20, 20-39, 40-59, and >60 pack-years. They suggest that FOT parameters have the potential to detect early smoking-induced respiratory alterations [16].

The study by Shinke et al. investigated FOT parameters in smokers with and without COPD. They observed that around 60% of smokers exhibited increased respiratory resistance and negative shifts in reactance, resembling patterns seen in COPD patients, suggesting early small airway remodeling. However, limitations include the lack of significant associations between impedance parameters and smoking history [17].

The 2017 comparative cross-sectional study by Schivinski et al. on healthy children and adolescents exposed to passive smoking revealed that they displayed lower spirometric values and higher impulse oscillometry (IOS) parameters compared to nonexposed individuals, indicating alterations in both forced and quiet parameters of lung function [18].

The multinational study by Crim et al. evaluated respiratory system impedance using IOS in COPD patients and control subjects, including current/former smokers and nonsmokers. IOS demonstrated differences in respiratory impedance between COPD patients and controls, though correlations with CT indices were poor [19].

Silva et al. conducted a Brazilian study examining FOT parameters in COPD patients across different GOLD stages, identifying significant differences in impedance parameters indicative of airway obstruction. The study demonstrated the diagnostic accuracy of within-breath FOT parameters in differentiating COPD patients from controls. However, limitations include the focus on respiratory impedance without a comprehensive analysis of other factors influencing COPD progression and the limited exploration of predictive endpoints [20].

The US study by Berger et al. investigated distal lung function using FOT in asymptomatic smokers at risk for COPD, highlighting correlations between abnormal FOT findings and increased lung inflammation through the study's cross-sectional design and small sample size, limiting generalizability [21].

Jetmalani et al. conducted an Australian study and assessed IOS parameters in young-to-middle-aged smokers with normal spirometry, revealing common abnormalities suggestive of peripheral airway dysfunction not captured by spirometry alone. Limitations include the lack of structural and functional imaging data to complement IOS findings and the focus on a specific age group, limiting generalizability [22].

Overall, the studies provide valuable insights into the utility of FOT and IOS in assessing respiratory mechanics in smokers. While these techniques show promise in detecting early changes in lung function and peripheral airway dysfunction, further research is needed to validate their clinical utility, establish longitudinal associations with disease progression, and explore their potential as predictive endpoints for COPD morbidity. Additionally, larger scale studies with diverse populations are warranted to enhance the generalizability of findings across different patient groups. The absence of significant differences in FOT parameters between different smoking indexes in the given populations can be attributed to several factors.

First, the relatively small sample size might lack the statistical power to detect subtle variations between the two groups. Methodological considerations such as variability in FOT equipment could also contribute to the lack of significant findings. We have included only healthy smokers in our study compared to other studies that recruited subjects with existing lung disease. Stratification of the subject based on the smoking index compared to pack year may have affected the result. Additionally, the cross-sectional nature of the study design imposes limitations on establishing causal relationships between smoking and respiratory parameters. Longitudinal studies would provide more robust evidence by tracking changes in respiratory function over time in response to smoking exposure. Finally, the reliance on self-reported smoking index and potential inaccuracies or underreporting of smoking behaviors may introduce measurement error, reducing the strength of observed associations.

It is crucial to interpret these results within the context of the study limitations and potential confounding factors. Further research with larger and more diverse populations is warranted to confirm these findings and explore potential underlying mechanisms. Additionally, longitudinal studies are needed to investigate the long-term effects of smoking on respiratory mechanics assessed by FOT, which could provide valuable insights into the progression of smoking-related respiratory diseases.

The Pearson correlation matrix presents the associations between the smoking index and various spirometry and FOT parameters. Overall, the correlations appear to be weak to moderate, with several statistically significant relationships observed.

Notably, there are significant negative correlations between airway resistance parameters (R5, R5-20) and FEV1/FVC ratio, indicating that higher airway resistance is associated with lower FEV1/FVC values, which is indicative of obstructive lung disease.

Airway resistance represents the resistance encountered by airflow as it moves through the airways during breathing. When airway resistance is elevated, as indicated by higher R5 and R5-20 values, it suggests that the airways are narrowed or obstructed, making it more difficult for air to flow in and out of the lungs efficiently.

The FEV1/FVC ratio is a measure of airflow limitation and is typically reduced in obstructive lung diseases. A lower FEV1/FVC ratio indicates that a larger proportion of the FVC is exhaled during the first second of forced expiration (FEV1), characteristic of obstructive lung function patterns.

Therefore, the negative correlation between airway resistance parameters and the FEV1/FVC ratio suggests that as airway resistance increases, the ability of the lungs to expel air rapidly during forced expiration decreases, leading to a lower FEV1/FVC ratio and indicating the presence of airflow obstruction.

Moreover, significant negative correlations are also identified between the area of reactance parameters (AX and Fres) and FEV1. This indicates that as the area of reactance increases, reflecting alterations in airway compliance and stiffness, the FEV1 decreases, indicating potential impairment in lung function. When the area of reactance is increased, it suggests reduced compliance or increased stiffness of the airways, which can impede airflow dynamics.

The decrease in FEV1 associated with increased area of reactance indicates that as airway stiffness or reduced compliance increases, the ability of the lungs to expel air during forced expiration diminishes. This reduction in FEV1 suggests potential impairment in lung function, which is consistent with the physiological changes seen in obstructive lung diseases where airway compliance is decreased due to inflammation, airway remodeling, or bronchoconstriction.

Overall, these physiological interpretations highlight the intricate relationship between airway mechanics and lung function parameters, underscoring the importance of assessing both airway resistance and reactance parameters in understanding respiratory physiology and diagnosing obstructive lung diseases.

Moreover, using Pearson correlation coefficients assesses linear relationships between variables but may not capture nonlinear or more complex associations. This limitation could obscure potential nonlinear relationships between the smoking index and respiratory parameters.

Overall, while the study provides valuable insights into the relationships between the smoking index and respiratory parameters, the findings should be interpreted cautiously due to the aforementioned limitations. Future research with larger, more diverse samples, longitudinal designs, and comprehensive assessments of smoking exposure and respiratory function would enhance our understanding of these associations.

## Conclusions

FOT, while sensitive to certain aspects of respiratory function, may not capture subtle changes induced by early smoking exposure. The marginal significance observed in FRES warrants further investigation, possibly indicating alterations in respiratory mechanics associated with longer durations of smoking. The observed correlations between respiratory parameters in the study tend to be weak to moderate in strength. This indicates that while some relationships exist, there are associations worthy of attention. Specifically, notable negative correlations are found between airway resistance parameters (such as R5 and R5-20) and the FEV1/FVC ratio, and significant negative correlations are also identified between airway reactance parameters (AX and Fres) and FEV1.
